# Improving workflow in prostate MRI: AI-based decision-making on biparametric or multiparametric MRI

**DOI:** 10.1186/s13244-021-01058-7

**Published:** 2021-08-09

**Authors:** Andreas M. Hötker, Raffaele Da Mutten, Anja Tiessen, Ender Konukoglu, Olivio F. Donati

**Affiliations:** 1 Institute of Diagnostic and Interventional Radiology, University Hospital Zurich, Rämistrasse 100, 8091 Zurich, Switzerland; 2 Computer Vision Laboratory, Department of Information Technology and Electrical Engineering, ETH Zurich, Sternwartstrasse 7, 8092 Zurich, Switzerland

**Keywords:** Multiparametric MRI, Prostate cancer, Artificial Intelligence

## Abstract

**Objectives:**

To develop and validate an artificial intelligence algorithm to decide on the necessity of dynamic contrast-enhanced sequences (DCE) in prostate MRI.

**Methods:**

This study was approved by the institutional review board and requirement for study-specific informed consent was waived. A convolutional neural network (CNN) was developed on 300 prostate MRI examinations. Consensus of two expert readers on the necessity of DCE acted as reference standard. The CNN was validated in a separate cohort of 100 prostate MRI examinations from the same vendor and 31 examinations from a different vendor. Sensitivity/specificity were calculated using ROC curve analysis and results were compared to decisions made by a radiology technician.

**Results:**

The CNN reached a sensitivity of 94.4% and specificity of 68.8% (AUC: 0.88) for the necessity of DCE, correctly assigning 44%/34% of patients to a biparametric/multiparametric protocol. In 2% of all patients, the CNN incorrectly decided on omitting DCE. With a technician reaching a sensitivity of 63.9% and specificity of 89.1%, the use of the CNN would allow for an increase in sensitivity of 30.5%. The CNN achieved an AUC of 0.73 in a set of examinations from a different vendor.

**Conclusions:**

The CNN would have correctly assigned 78% of patients to a biparametric or multiparametric protocol, with only 2% of all patients requiring re-examination to add DCE sequences. Integrating this CNN in clinical routine could render the requirement for on-table monitoring obsolete by performing contrast-enhanced MRI only when needed.

**Supplementary Information:**

The online version contains supplementary material available at 10.1186/s13244-021-01058-7.

## Key points

- AI helps in automated decision-making between biparametric and multiparametric prostate MRI protocols.
- AI would have correctly assigned 78% of patients to a biparametric/multiparametric protocol.
- Re-examinations would have only been necessary in 2% of all patients.
- The performance of the trained network differed slightly between MRIs from different vendors.

## Background

Prostate MRI has shown considerable clinical value in detection and staging of prostate cancer and is part of clinical routine in most institutions worldwide [1–4]. Conventionally, “multiparametric” prostate MRI consists of high-resolution *T*2—weighted, diffusion- weighted and dynamic contrast-enhanced (DCE) sequences [5]. Recently, the use of an abbreviated MRI protocol without application of a contrast agent (termed “biparametric MRI”) has been proposed and several investigations have reported a comparable performance of the protocol in cancer detection compared to the complete multiparametric protocol [6–11]. Omittance of DCE-MRI from the acquisition results in shorter examinations, an optimization duly needed in times of increasing demand for prostate MRI. Furthermore, biparametric MRI of the prostate avoids any contrast agent side effects, improves cost-effectiveness, and optimizes general workflow in the radiology department. In addition, DCE sequences are deemed of diagnostic quality in only a subset of patients, as recently reported by the PRECISION study group [12]. However, as DCE-MRI is known to reduce the number of indeterminate lesions, and to be of particular value in examinations with poor image quality or for the less-experienced radiologist [5, 13, 14], on-table monitoring and an individualized per-patient decision on DCE are currently proposed by the PI-RADS committee [15].

Ideally, the decision to inject a contrast agent should therefore be performed by an experienced radiologist on a per-scan and ad-hoc basis, and its application should be limited to those cases when it is deemed to improve clinical decision-making. However, given the expected rise in examinations due to the inclusion of prostate MRI into national and international urologic guidelines, such an individual and timely decision on every prostate MRI may not be feasible anymore in a clinical setting for most institutions.

Therefore, we sought to develop, train, and validate convolutional neural network (CNN) that would automatically identify patients in whom acquisition of a DCE sequence would be beneficial. This would allow for shorter biparametric examinations for many patients and increase patient safety by the omission of contrast media injection while simultaneously avoiding a decreased diagnostic accuracy in those patients who would benefit from a complete multiparametric MRI protocol.

## Materials and methods

### Patient cohorts and image analysis

This study was approved by the institutional review board and the requirement for study-specific informed consent was waived. A retrospective search was performed on our prospectively maintained institutional database from 02/01/2018 to 11/30/2019 for consecutive patients undergoing multiparametric prostate MRI (in accordance with PIRADS guidelines [5]) for suspicion of prostate cancer. Three distinctive cohorts were formed: (1) a group of 300 multiparametric prostate MRI for training of the neural network (“training set”) (see Additional file 1: Appendix S1), (2) a group of 100 multiparametric prostate MRI for validation of the trained network (“validation set”) and (3) a group of 31 patients undergoing prostate MRI on a scanner from a different vendor (“different vendor set”). The MRI examinations included into the “training” and “validation” were performed in 363 patients (age at time of MRI: 64.4 years, mean PSA at time of MRI: 8.33 ng/ml). All MRI scans of the first two groups were performed on Siemens Skyra scanners (Siemens Healthineers, Erlangen, Germany) at a field strength of 3 Tesla using a 60 Ch or 18 Ch phased-array body coil, while the “different vendor set” underwent examinations on a GE Discovery MR750w (GE Healthcare, Chicago, Illinois, USA) using a 16 Ch phased-array body coil at a field strength of 3 Tesla. Typical MRI parameters for axial *T*2—weighted and diffusion-weighted sequences can be found in Additional file 1: Table S1. Two board-certified radiologists with 10 and 7 years of experience in dedicated prostate imaging (‘expert radiologists’ according to ESUR/ESUI consensus [16], O.F.D. and A.M.H., *R*1 and *R*2) independently reviewed all examinations of the “training set”, “validation set” and “different vendor set” and scored whether DCE sequences would have been beneficial for diagnosis (regardless of the reason, e.g. distortions by rectal gas, low signal-to-noise-ratio etc.). After completion of readings a consensus was reached by the two readers by reviewing all examinations with discrepant decisions. The resulting consensus on the “training set” was used as reference standard for training of the CNN. In addition, the “validation set” was reviewed by a technician (*R*3) with daily practice in acquiring prostate MRI examinations and again the necessity of DCE sequences was noted.

### Training and validation of a neural network

A detailed account including technical specifications of the neural network can be found in Additional file 1: Appendix S1. The accompanying PyTorch code and the training scheme can be found at https://github.com/enderkon/ProstateQC.git.

**Fig. 1:**
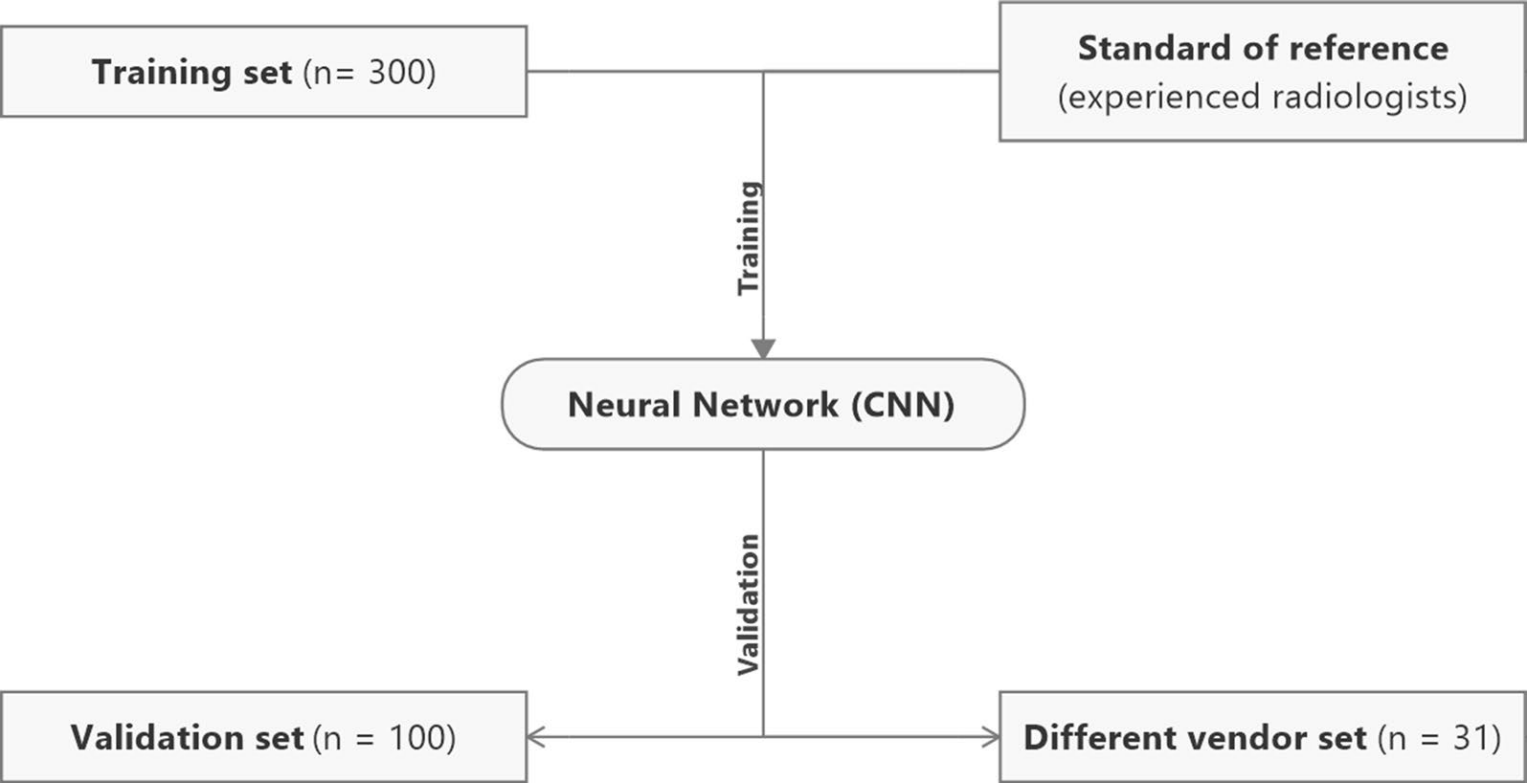
Flow chart detailing the process of training and validation of a neural network for deciding on the necessity for DCE sequences. Consensus of two experienced radiologists was standard of reference for all comparisons

Development of the convolutional neural network was based on *T*2—weighted axial images and corresponding diffusion-weighted images (*b* values of 100, 600 and 1000 s/mm^2^). After exporting the anonymized image data of these sequences from PACS, images were pre-processed to standardize pixel size across all images. The convolutional neural network was trained on a set of 300 MRI examinations (“training set”) with the consensus on desirability of DCE by two experienced radiologists as reference standard and then applied to a separate set of 100 MRI examinations not used for training (“validation set”). Separate branches for anatomical (*T*2—weighted) and diffusion-weighted images were created, whose outputs were then reduced to receive a single probability (ranging from 0 to 1, with 0 meaning “DCE not desirable” and 1 meaning “DCE highly desirable”). For training, 10 random experiments were performed with the training set being split into training (80%), validation (5%) and testing partitions (15%) randomly, with the training partition being used to train the network, the validation partition being used to decide when to stop training and testing partition to monitor the error. The model was trained for 30 epochs for each random experiment, the iteration that led to the lowest classification error and cross-entropy loss on the validation set was determined to be the final model for experiment. While analyzing validation data, all 10 trained networks were applied separately, and results were aggregated through averaging to yield the final prediction for each image. The trained network was consecutively validated in a training set with images of 100 additional patients to evaluate its sensitivity and specificity and in a separate set of 31 prostate MRIs performed on a scanner from a different MRI vendor, see Fig. 1.

### Statistics

To assess inter-reader agreement, Cohen’s kappa was estimated and interpreted as proposed by Landis and Koch [17] and as follows: excellent agreement > 0.75, good agreement 0.59–0.75, fair agreement 0.40–0.58, poor agreement < 0.4. Diagnostic accuracy was assessed by the area under the curve of a receiver-operator-characteristics (ROC) analysis and the best cut-off value was estimated by maximizing the Youden index.

All statistical analyses were performed using IBM SPSS Statistics 26 (IBM Inc., Armonk, USA) and MedCalc 18.2.1 (MedCalc Software Ltd, Ostend, Belgium).

## Results

### Inter-reader agreement

*R*1 and *R*2 agreed on the necessity for DCE sequences in 267/300 (89%) cases of the “training set” (kappa: 0.76), 89/100 (89%) cases of the “validation set” (kappa: 0.76) and in 26/31 cases of the “different vendor set” (kappa: 0.64). In the remaining examinations, a consensus reading was needed to complete the standard of reference. Agreement between *R*3 (technician) and the reference standard (consensus of *R*1 and *R*2) for the “validation set” was fair with a kappa of 0.55 (see Table 1).

**Table 1:** Performance of reader 3 (technician) and the artificial intelligence in correctly deciding on the necessity of contrast injection in the validation set (*n* = 100) with the consensus of two experienced radiologists (*R*1 and *R*2) as reference standard.

	DCE necessary	DCE not necessary	Agreement	AUC (95% CI)	Sensitivity	Specificity
Consensus (*R*1/*R*2)	36/100 (36%)	64/100 (64%)	Ref	Ref	Ref	Ref
Technician (*R*3)	70/100 (70%)	30/100 (30%)	0.55	0.765 (0.669–0844)	63.9%	89.1%
Artificial Intelligence (AI)	56/100 (56%)	44/100 (44%)	0.54	0.881 (0.801–0.937)	94.4%	68.8%

Agreement: kappa with Consensus as reference standard; AUC: Area-under-the-curve; Sensitivity and Specificity of the artificial intelligence based on ROC analysis with a maximized Youden index and high sensitivity to avoid re-examinations

### Diagnostic accuracy of the neural network

**Fig. 2:**
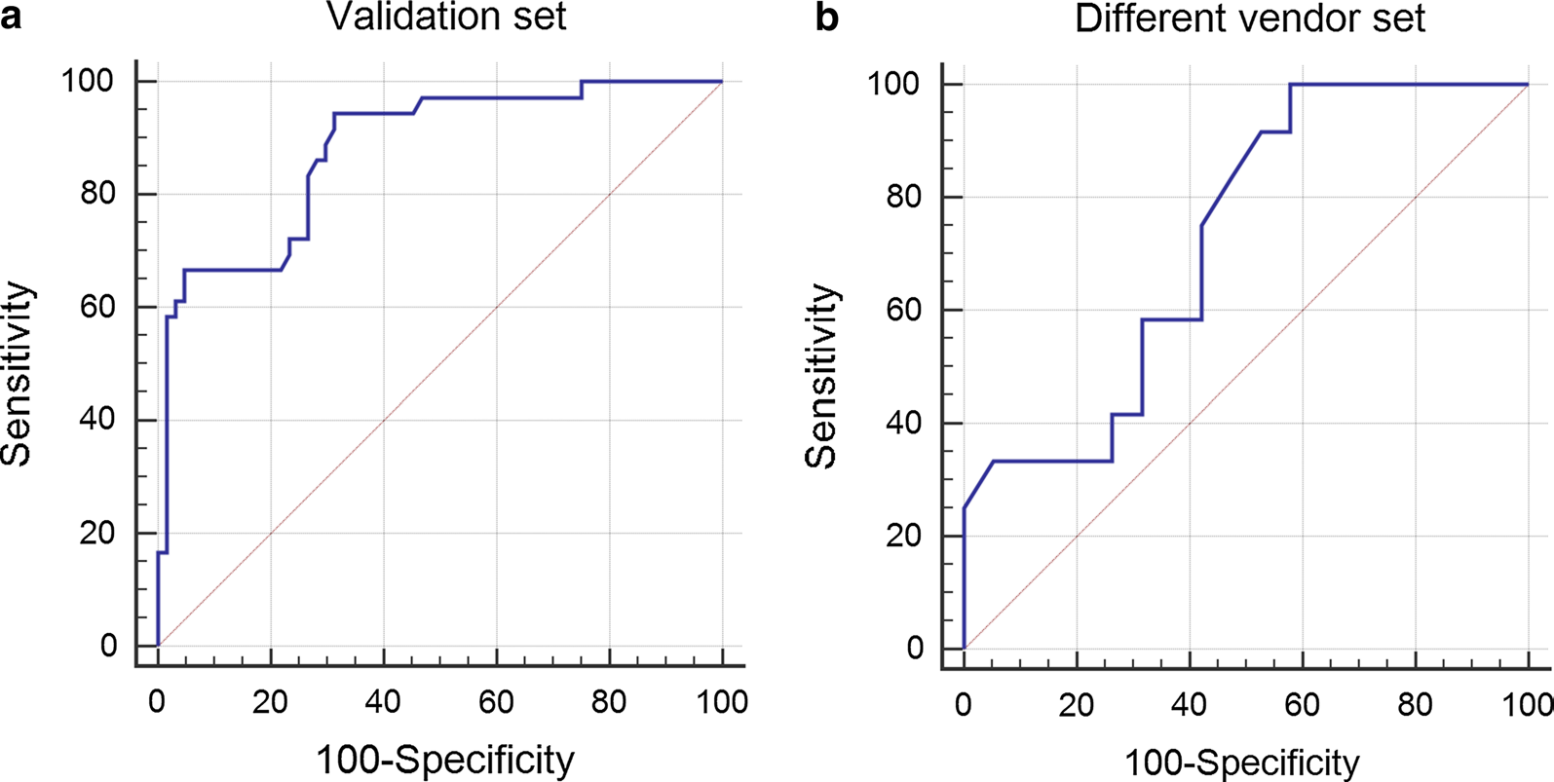
Receiver-Operating-Characteristic-Curves (ROC-Curves, numbers are percentages) of the diagnostic performance of the convolutional neural network in the “validation set” (**a** *n* = 100, AUC: 0.88) and “different vendor set” (**b** *n* = 31, AUC: 0.73).

The final neural network showed a sensitivity of 94.4% and specificity of 68.8% in the “validation set” when maximizing the Youden index (AUC: 0.88, J: 0.63) in ROC analysis (see Fig. 2a). When aiming for a low rate of false negatives (a low re-examination rate for adding DCE sequences), this would result in 2% of all patients (and 2/36 patients with a need for DCE, 5.6%) needing a supplementary examination including the injection of a contrast agent (false negatives), while 44% of patients correctly underwent biparametric and 34% of patients correctly underwent multiparametric MRI (see Table 2). In 20% of patients, the CNN decided to perform DCE while the radiologists did not deem DCE to be necessary (false positives).

**Table 2:** ROC analysis results with maximized Youden index from the validation set (n = 100)

	Radiologists: DCE necessary	Radiologists: DCE not necessary
AI: DCE necessary	34/100 (34%)	20/100 (20%)
AI: DCE not necessary	02/100 (2%)	44/100 (44%)
	Sensitivity: 94.4%	Specificity: 68.8%

“DCE necessary/not necessary” is based on the consensus of two expert radiologists

*AI* Artificial Intelligence

**Fig. 3:**
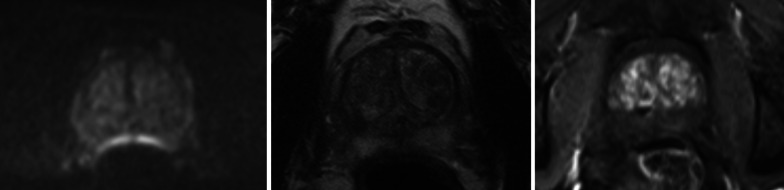
66 year-old patient undergoing prostate MRI for suspicion for prostate cancer. The presence of rectal gas results in susceptibility artifacts, distorting the diffusion-weighted image (left: DWI, *b* = 1000 s/mm^2^; middle: *T*2—weighted). Both the experienced radiologists and the AI opted for contrast, while reader 3 (technician) did not. Dynamic contrast-enhanced MRI (right) was helpful in ruling out any index lesions at this level of the prostate

With *R*3 (technician) reaching a sensitivity of 63.9% and specificity of 89.1%, the use of the neural network would allow for an increase in sensitivity of 30.5% at an albeit lower specificity (see Figs. 2b and 3).

When applying the trained neural networks to a set of MRI examinations from a different vendor, ROC analysis with maximized Youden index (AUC: 0.73, J: 0.42) demonstrated a sensitivity of 100% and a specificity of 42.1% (see Table 3).

**Table 3:** Performance of the trained neural network in the validation set and in the set with scans from a different vendor (ROC analysis; AUC: Area-under-the-curve, Criterion: associated criterion) with consensus from two experienced radiologists as standard of reference

	AUC (95% CI)	Criterion	Sensitivity (%)	Specificity (%)
AI validation set	0.881 (0.801–0.937)	> 0.221	94.4	68.8
AI different vendor set	0.726 (0.537–0.870)	> 0.171	100.0	42.1

## Discussion

Due to its necessity in order to perform targeted biopsies, the widespread integration of prostate MRI in the diagnostic workup of patients with suspected prostate cancer will likely lead to an increased number of examinations to be performed by radiology departments in the near future [1]. This represents a challenge, as it not only requires improving the radiological workflow [18], but also ensuring optimal image quality of the examinations, as stressed by the recently published PI-QUAL scoring system from the PRECISION trial group [12, 19]. We developed and validated a CNN to independently decide on the necessity of dynamic contrast-enhanced sequences (DCE) with high accuracy and with a very low false negative rate (i.e. a low rate of patients who falsely did not undergo DCE).

Multiparametric prostate MRI includes *T*2—weighted, diffusion-weighted and dynamic contrast-enhanced sequences. Recently, several authors suggested that dynamic contrast-enhanced sequences could be omitted from the MRI protocol (“biparametric MRI”), thus shortening examination times and avoiding any potential unwanted side effects from the contrast agent [6, 7]. However, it is also known that contrast-enhanced sequences can be of value in a subset of patients undergoing prostate MRI [20, 21] and that they can be of particular value for the unexperienced radiologist and in examinations with artifacts (e.g., from hip prothesis) or poor image quality [14]. While previous papers focused on the use of AI in prostate MRI to improve planning and image quality of the examinations [18] or tumor detection [22, 23], we aimed to harness the benefits of artificial intelligence to improve quality control and workflow-relevant decision making.

Ideally, the decision on whether to perform DCE should be made on a per-patient and ad-hoc basis, as currently proposed by the PI-RADS committee [15]. However, applying on-table monitoring and having a radiologist render this decision on a per-case basis oftentimes is not feasible in clinical routine—and re-examinations should be avoided particularly to not endanger the (time) benefits gained from not performing DCE sequences in every patient. In this study we sought to delegate the task of deciding between a biparametric and multiparametric protocol to artificial intelligence, which would allow for real-time decision-making and a straightforward implementation into the clinical workflow. The trained neural network was able to correctly decide on contrast agent application with a very high sensitivity of > 94%. This approach would have correctly assigned 44% of patients to a biparametric protocol—thus sparing them from contrast injection—and 34% to a standard multiparametric MRI. Twenty percent of patients would have undergone multiparametric instead of biparametric MRI based on the algorithm’s decision. At the same time, only 2% of all patients (5.6% in the subgroup of patients with the need for DCE) would not have received DCE when expert radiologists would have deemed it necessary. Depending on the clinical question posed in these patients, they could be scheduled for a re-examination. Finally, performance of the neural network was superior to the accuracy of the radiology technician acquiring the images (reader 3 in this study), i.e. it would be beneficial having the neural network deciding on DCE in clinical routine. This would particularly apply in a setting where the biparametric MRI protocol is used as an institutional standard for detection of target lesions on prostate MRI. The AI could automatically detect a scan that might require the acquisition of DCE sequences with high accuracy and alert the attending radiologist (who still has to supervise the application of contrast agent due to legal reasons and the possibility for adverse reactions).

When applying the neural network trained on in-house scanners to a set of MRI examinations performed on a different scanner, a sensitivity of 100% at an albeit lower specificity of 42.1% was achieved. While this is certainly an encouraging result and shows that the network is not restricted to images from scanners of a certain manufacturer, a dedicated training set based on scans from this different vendor would likely further increase the accuracy of the neural network.

In addition, our approach could be improved and refined in a few ways: Though the testing set for the neural network consisted of sequentially acquired clinical MRI examinations, the artificial intelligence requires validation in clinical routine in the future. In this study, the decision rendered by the neural network was regarded as dichotomous—however, it would be possible to define a range of probability values in which the AI is unsure in its decision, which would prompt the technician to call a radiologist for this particular examination. This approach could reduce the number re-examinations, while still allowing for omittance of DCE in many cases. Also, the consensus of two “expert level” radiologists was used as standard of reference in this study. However, there might be cases in which a more novice reader would have appreciated a DCE sequence when the more experienced reader does not require it. In addition, while we assessed the performance of the neural network in a set of MRI examinations from a different vendor, the number of scans included into the “different vendor set” was rather low. However, as the code for the neural network will be freely available, our results can easily be tested in a different institution and with different scanners.

In conclusion, we designed a neural network with the ability to accurately decide between acquisition of a full multiparametric MRI protocol including DCE and a faster biparametric protocol. The rate of patients who would have left the MRI scanner without an ultimately needed DCE sequence based on the decision made by the CNN was very low. Hence, integration of AI into quality assessment and decision making could allow for shorter examination times and a more streamlined clinical workflow, while maintaining diagnostic accuracy by including DCE only when truly needed.

## Supplementary Information

**Additional file 1**. Technical details on the implementation of the neural network.

## Data Availability

A detailed account including technical specifications of the neural network can be found in Additional file 1: Appendix S1. The accompanying PyTorch code and the training scheme can be found at https://github.com/enderkon/ProstateQC.git.

## Abbreviations

AI: Artificial intelligence
AUC: Area-under-the-curve
CNN: Convolutional neural network
DCE: Dynamic contrast-enhanced
DWI: Diffusion-weighted imaging
mpMRI: Multiparametric MRI
PI-RADS: Prostate imaging reporting and data system
ROC: Receiver-operating characteristics
