# Supplementary material for: Improving workflow in prostate MRI: AI-based decision-making on biparametric or multiparametric MRI
Source: Insights Imaging. 2021 Aug 9;12:112. doi: 10.1186/s13244-021-01058-7 (PMC8353049; doi:10.1186/s13244-021-01058-7)
Supplement: Supplementary file 1 — Additional file 1. Technical details on the implementation of the neural network. [file 13244_2021_1058_MOESM1_ESM.docx]

Supplementary Appendix 1: Technical specifications

**Data Pre-processing**

In order to standardize the images and facilitate training an algorithm, we resized and cropped/padded each image according to sequence-specific values. We first determined minimum pixel-sizes for each sequence across the training set. All images of the same sequence were resampled at the minimum pixel size. Only in-plane resizing was used. Through-plane resolutions were very similar across the images; hence no resizing was performed. There are two things to note here: First, it is common practice to use maximum pixel-size across the dataset for normalizing pixel-sizes. However, as the goal of the project was to determine image quality, we used the minimum pixel-size in this project. Images with high pixel-sizes may have lower quality than those with lower pixel-sizes. To avoid losing this possible source of information, we upsampled images whenever necessary instead of downsampling. Upsampling introduces interpolation artifacts in the images, however, such artifacts would not decrease the quality of the original scan, therefore not interfere with the purpose. Downsampling, however, would have decreased the quality of the original scan. Second, we did not normalize through-plane resolution. All images had similar through-plane resolutions. Normalizing for the small variations can substantially reduce the quality of the in-plane scans, which are used for diagnostic purposes.

Following the resizing, we cropped or padded with zeros each image according to the minimum sequence-specific field of view also computed using the training set. For each sequence we identified the minimum field-of-view and processed each sample in the dataset to have exactly the same field-of-view. Having the same pixel-size, this leads to having the same image size. After resizing and cropping / padding, we end up with a dataset where images from different sequences have the same size and pixel-size.

The sequence-specific pixel and image sizes are provided in the tables below.

| **Sequence** | **Dx** | **Dy** | **Dz** |
| --- | --- | --- | --- |
| T2 axial | 0.25 | 0.25 | 3.0 |
| T2 sagittal | 0.42 | 0.42 | 3.0 |
| T2 coronal | 0.25 | 0.25 | 3.0 |
| DWI | 0.8 | 0.8 | 3.0 |

***Table 1:*** *Pixel size that was used for the experiments. All images were resampled to achieve the same pixel size. Different pixel sizes were used for different sequences as indicated in the table.*

*.*

| **Sequence** | **Image Size - X** | **Image Size - Y** | **Image Size - Z** |
| --- | --- | --- | --- |
| T2 axial | 639 | 639 | 21 |
| T2 sagittal | 383 | 383 | 17 |
| T2 coronal | 639 | 639 | 21 |
| DWI | 97 | 104 | 17 |

***Table 2:*** *Image size used in the experiments. All images are cropped or padded with zeros to achieve the indicated image sizes. Different image sizes were used for different modalities. In addition, the DWI sequence included three different trace images obtained with b-values of 100, 600 and 1000 s/mm².*

**Cropping T2-weighted axial images**

In order to avoid possible overfitting, we reduced the information before feeding images to a neural network by focusing on an ROI encapsulating the prostate. We used the FOV in the sagittal and coronal acquisitions of T2-weighted images to determine an ROI in the axial acquisitions. The ROI covered the entire prostate as well as a small region around the prostate; most of the anatomical variation that we believe not to be related to the decision of acquiring DCE is cropped out. The cropped T2-weighted axial ROI accompanies the DWI images, which are also acquired around the same ROI.

The 300 training samples, 100 validation samples and 31 samples from the different vendor have all be pre-processed and normalized the same way. The minimum pixel-sizes and field-of-view, which are shown in the tables, were determined using only the training set.

**Network model**

For each subject, we had access to their axial T2-weighted and diffusion weighted images. We use both sources of data to predict whether an additional dynamic contrast imaging will be required. This could create a memory problem for neural network training as well as substantially increase the number of parameters required to process all the possible sequences as different channels.

We therefore used two different assumptions to reduce the size of the input and address both memory problems as well as reduce the number of required parameters. First, the model only uses the cropped T2-weighted ROIs in the axial acquisitions. In deciding whether a dynamic contrast image is required, experts mostly refer to the axial structural acquisition. Second, we only utilize trace images computed from diffusion acquisitions using three different b-values. Expert opinion is that these images provide all the necessary information to decide whether a DCE image is required or not. Following this observation, the network only uses the three DWI images acquired at the mentioned b-values. Based on these two assumptions we reduce the network input to only 4 volumetric images.

We used a 3D convolutional neural network (CNN) with two branches, one for structural acquisitions and one for DWI images, whose outputs are reduced using global average pooling and concatenated before a final fully connected linear layer that outputs a probability. The probability indicates the necessity to require a DCE image, with a value of 1 meaning that a DCE image is necessary while a value of 0 means that it is not required. Each branch outputs a vector of size 32. When concatenated this yields a vector of size 64 that summarizes the information in anatomical and DWI images. This vector is fed into a linear layer followed by a logistic function to get a single probability. Each branch can be represented as follows:


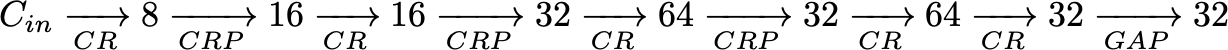


where numbers represent the number of channels, *CR* represents 3D convolution with kernel size 3x3x3 followed by rectified linear unit activation, *CRP* represents an additional pooling layer that decreases the channel size by half and *GAP* represents the global average pooling layer that takes the average within channels. The network’s input is *C_in_ =* 1 for T2-weighted anatomical images and 3 for diffusion images. The first *CR* and *CRP* layers at the beginning of each branch do not use padding and the pooling reduces the channel size in all three dimensions. Remaining layers use padding in the inferior-superior direction and pooling layers only reduce the channel size in the axial plane.

The outputs of the GAP layers in both branches are 32-dimensional vectors. They are concatenated to form a 64-dimensional vector that is fed into the final fully connected layer. The motivation for using a GAP at the end is to reduce the number of required parameters for a fully connected layer. Both branches as well as the final fully connected layer are trained end-to-end to reduce a cross-entropy loss between network’s output and ground-truth that indicates for each sample whether a DCE image is required based on consensus of two expert radiologists whose domain of expertise is prostate imaging.

We provide a PyTorch code for the network as well as the training scheme along this article.

|  | Siemens | | GE | |
| --- | --- | --- | --- | --- |
|  | T2-weighted | Diffusion-weighted | T2-weighted | Diffusion-weighted |
| TR (ms) | 7700 | 6400 | 7300 | 4000 |
| TE (ms) | 92 | 81 | 123 | 73 |
| In-plane resolution (mm) | 0.25 x 0.25 | 0.8 x 0.8 | 0.39 x 0.39 | 0.7 x 0.7 |
| Number of averages | 3 | 2, 4, 8 | 1.5 | 2, 6, 16-18 |
| Slice thickness (mm) | 3 | 3 | 3 | 3 |
| b-values (s/mm²) | - | 100, 600, 1000, 1400 (calculated) | - | 50/100, 600, 1000, 1400 (calculated) |

**Supplementary Table 1**: Typical MRI parameters for axial T2-weighted and diffusion-weighted sequences for both vendors.
